# Association between fish consumption and sleep disorders among Chinese adults: a cross-sectional study

**DOI:** 10.1080/07853890.2025.2491663

**Published:** 2025-04-20

**Authors:** Siyuan Li, Mingwei Ma, Liming Hu, Jiaying Lao, Xingguang Luo, Jie Pan, Dafeng Lu, Min Wang, Wenhui Lin, Yuncao Fan, Fan Wang, Yu-Hsin Chen, Penghui Wang, Fenzan Wu, Xiaojie Wei, Jinzhong Xu, Yanlong Liu, Lin Zheng

**Affiliations:** a Department of Clinical Pharmacy, Affiliated Wenling Hospital, Wenzhou Medical University, Wenling, China; b School of Mental Health, Wenzhou Medical University, Wenzhou, China; c Department of Psychiatry, Yale University School of Medicine, New Haven, CT, USA; d Quzhou Center for Disease Control and Prevention, Quzhou Center for Public Health Service, Quzhou, China; e Department of Cardiovascular Medicine, Affiliated Wenling Hospital, Wenzhou Medical University, Wenling, China; f Beijing Hui-Long-Guan Hospital, Peking University, Beijing, China; g Affiliated Cixi Hospital, Wenzhou Medical University, Ningbo, China

**Keywords:** Fish intake, sleep disorders, sleep quality, insomnia

## Abstract

**Purpose:**

This study aimed to investigate the potential influence of fish consumption on sleep disorders and their specific dimensions among adults in China.

**Methods:**

A cross-sectional study was conducted involving 904 participants aged 28–95 from Wenling, China. Fish intake was assessed using a Food Frequency Questionnaire containing 10 items. Sleep quality was evaluated using the Insomnia Severity Index (ISI) and Pittsburgh Sleep Quality Index (PSQI). Participants were categorized into three groups based on weekly fish intake. Logistic regression analyses were employed to determine the association between fish intake and the prevalence of sleep disorders and their specific dimensions.

**Results:**

Higher marine fish intake was negatively associated with PSQI subdimensions daytime dysfunction, sleep latency and sleep quality scores compared to lower fish intake (adjusted odds ratio (OR): 0.316, 95% confidence interval (CI): 0.205–0.486; adjusted OR: 0.462, 95% CI: 0.302–0.706; and adjusted OR: 0.568, 95% CI: 0.369–0.861, respectively). Marine fish consumption appears to have a positive association with sleep quality, as well as short sleep latency and daytime functioning, among adults in China.

**Conclusions:**

This study provides novel insights into the association between fish intake and sleep disorders and their specific dimensions.

## 1. Introduction

Sleep disorders pose a significant challenge to both mental and physical health globally, with prevalence rates ranging from 3.9% to 45.0%, leading to heightened risks of conditions such as obesity, diabetes, cardiovascular diseases, cognitive impairments and mental illnesses [1–5]. Among sleep disorders, insomnia stands out as a major concern [6], affecting more than one-third of adults at some point in their lives. Current therapeutic approaches, including benzodiazepines, melatonin and cognitive-behavioural therapy, offer only modest efficacy in clinical settings [7,8]. In recent years, lifestyle factors have emerged as crucial determinants of sleep, offering a novel perspective [9]. Mounting evidence suggests that dietary habits, in particular, play a pivotal role in improving sleep. Studies indicate that a healthy dietary pattern, characterized by high intake of vegetables, fruits and fatty fish, can alleviate sleep problems [10,11]. Furthermore, specific nutrients present in foods, such as magnesium, zinc and omega-3 fatty acids, have been linked to the modulation of sleep patterns [12,13]. Additionally, the association between individual food items and sleep quality has garnered attention [14,15].

In Chinese traditional diets, seafood, particularly marine fish like hairtail and yellow croaker, holds a significant place, being rich in protein, polyunsaturated fats and essential vitamins. Fish is known to contain high levels of melatonin, a hormone produced by the pineal gland [16], which plays a crucial role in regulating sleep–wake rhythm and related disorders [17–19]. Exogenous melatonin supplementation has positive effects on sleep quality in a meta-analysis [20]. The two key pineal biochemical functions, lipoxygenation and melatonin synthesis, may be synergistically regulated by the status of n-3 essential fatty acids [21]. Therefore, the chronic deficiency in melatonin production may be associated with reduced sleep quality in humans [22]. Fish and fish oils, as primary sources of omega-3 polyunsaturated fatty acids contain eicosapentaenoic acid (EPA) and docosahexaenoic acid (DHA) that regulate various brain functions [23,24]. Moreover, they act as circadian rhythm synchronizers that participate in a variety of physiological functions [25], positively impacting pineal function and therefore influencing sleep quality [26]. Specifically, omega-3 polyunsaturated fatty acids are known regulators of serotonin release and serotonin receptor function, which is converted from tryptophan by tryptophan hydroxylase 2 in the presence of active vitamin D hormone in the brain [27]. As such, the consumption of marine fish not only provides high-quality proteins, essential amino acids (e.g. tryptophan) but also important vitamins including vitamins A, D, E and K that aid in the regulation of an individual’s sleep and overall well-being [14,28,29]. Clinical studies have indicated higher expression of vitamin D receptors in brain regions involved in sleep regulation, suggesting a potential role of vitamin D in sleep modulation [30]. Notably, research demonstrates that fish consumption can indeed enhance sleep quality and daytime function [14,31]. Furthermore, a randomized controlled trial has shown that consuming oily fish can reduce sleep latency and daytime dysfunction in children [32]. Despite these findings, limited research has explored the effects of fish consumption on specific dimensions of sleep.

Therefore, this study aimed to investigate whether marine fish consumption could improve sleep quality and specific indicators of sleep disorders among adults. The research objectives were twofold: (1) to explore the association between marine fish consumption and sleep quality and (2) to investigate the specific sleep disorder indicators influenced by fish consumption.

## 2. Materials and methods

### 2.1. Study design

This cross-sectional analysis aimed to assess the relationship between dietary patterns, lifestyle and diseases among residents of Taizhou, Zhejiang Province, China. Participants were recruited from Wenling Hospital affiliated with Wenzhou Medical University. Trained investigators administered a comprehensive questionnaire covering sociodemographic characteristics, lifestyle variables, medical history and drug use. Laboratory results and special examinations were also collected.

### 2.2. Participants

**Figure 1.**
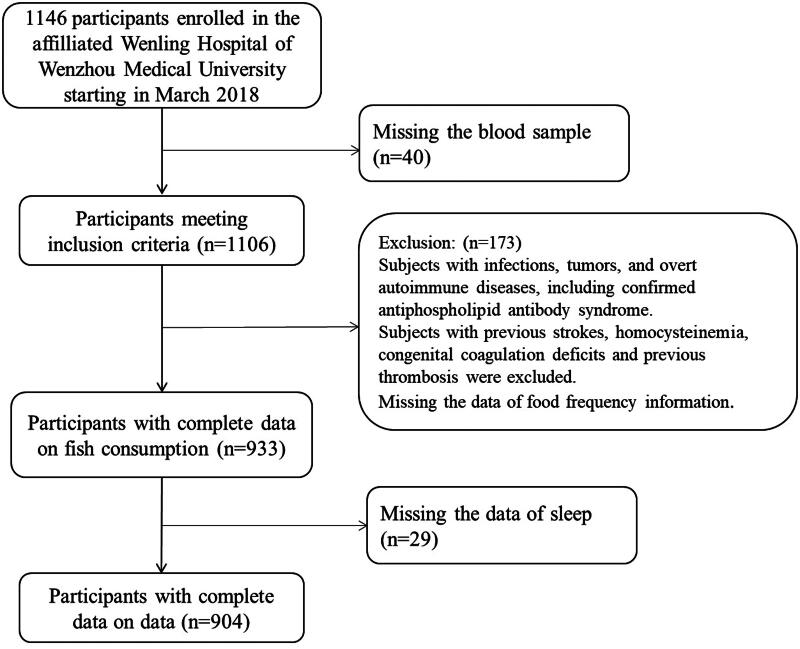
Flowchart of study participant selection.

Based on the initial proposal submitted to the institutional review board (IRB), the first phase of the proposal would prospectively recruit 1500 general patients who made scheduled visits to the cardiovascular medicine department starting in March 2018. Recruitment prematurely stopped due to the COVID outbreak and mandated lockdown, resulting in a total of 1146 patients, aged 25–95, prospectively recruited in this study. Participants in this study were individuals diagnosed with clinical manifestations of suspected cardiac artery disease and planned to undergo coronary angiography (CAG) and/or percutaneous coronary intervention (PCI) in Wenling Hospital affiliated with Wenzhou Medical University. Accordingly, 904 participants were included in this study after excluding the subjects with severe infections, tumours, overt autoimmune diseases, mental disorders and missing data (for details see Figure 1).

### 2.3. Clinical measurements

Trained medical staff collected anthropometric, demographic data, as well as FFQ, PSQI and ISI data from the participants using standard protocols after the individual was admitted to the hospital for CAG and/or PCI procedure. Demographic information included age, gender, height, weight and body mass index (BMI). Historical information, such as smoking status, alcohol consumption and medical history (hypertension, coronary heart disease, diabetes), was obtained through a standard questionnaire. Detailed measurement protocols are described elsewhere [15].

### 2.4. Dietary assessment

Informed by previous studies on food consumption in mainland China, such as those by Luo et al. [33], and Zhuang et al. [34], and considering the unique dietary characteristics of the Wenling populace, this study utilized a 10-item Food Frequency Questionnaire (FFQ). This instrument was designed to evaluate the average frequency of food and beverage consumption among participants over the past year, reflecting their personal dietary habits and lifestyle. The assessment was conducted using a five-point scale for consumption frequency, categorized as follows: ≤1 time per month, ≤1 time per week, <1 time per day, 1 time per day or ≥2 times per day. Items include milk (primarily liquid cow’s milk), salt, fruits, vegetables, red meat, seafood (primarily marine fish), eggs, soy products, nuts and sugar-sweetened beverages [33–36]. The dietary frequency questionnaire (FFQ) used in this study has been validated in a previous study [15]. The FFQ was conducted in Wenling City, Zhejiang Province, which is home to a national fishing port. The primary fish consumed locally include eels, yellow croaker, hairtail, knife fish and other marine species that are rich in EPA/DHA, with combined EPA and DHA values exceeding 0.5 g/100 g, thus meeting the definition of fatty fish [37–41]. According to that standard, the marine fish collected from individuals refers to eel, croaker, hairtail and saury in this study and they could belong to the ‘fatty fish’ category.

### 2.5. Sleep quality

The Pittsburgh Sleep Quality Index (PSQI) was employed to assess the sleep quality of individuals in the past month. The PSQI is a widely recognized tool for evaluating sleep quality and identifying sleep disorders in both research and clinical environments. Its validity and reliability have been well-established through previous studies [42,43]. The PSQI contains seven distinct domains: subjective sleep quality, sleep latency, sleep duration, habitual sleep efficiency, sleep disturbances, use of sleeping medication, and daytime dysfunction (for details see [42,43]). Each domain is scored on a scale from 0 to 3, with higher scores reflecting more severe issues. A cutoff point of 5 or higher based on the global score suggests the presence of poor global sleep quality [15,44]. The Insomnia Severity Index (ISI) is a well-validated scale consisting of seven items scored on a five-point Likert scale, with scores categorized into four levels of insomnia severity [45]. The recommended cutoff point in previous studies is a score below 8, suggesting little to no presence of clinical insomnia [46,47]. Poor sleep quality was defined as a PSQI global score greater than 5. Low subjective sleep quality was characterized by a PSQI subdimension score of 2 or higher, reflecting a self-reported perception of ‘fairly bad’ or ‘very bad’ sleep. Long sleep latency was defined as a period exceeding 30 min to fall asleep. Low habitual sleep efficiency was identified as a ratio of total sleep time to time spent in bed below 85%. Short sleep duration was defined as sleeping less than 7 h per night. Sleep disturbances were indicated by the occurrence of subjective sleep-related issues within the preceding month. The use of sleeping medication referred to the consumption of sleep aids during the previous month. Daytime dysfunction was defined as the experience of impaired daytime functioning within the past month [15,42].

### 2.6. Statistical analysis

Continuous data are presented as mean ± SD for normally distributed variables and median ± IQR for skewed variables. Categorical data are presented as numbers and percentages. Differences between groups were analysed using the Kruskal–Wallis rank sum test and chi-square test. Preliminary analyses of the data revealed, subjects either ate fish on a daily basis (≥7 per week), or they ate fish often (2–6 per week), or they rarely ate fish (≤1 per week). According to the data distribution, fish intake was divided into three tertiles (T1: ≤1 per week; T2: 2–6 per week; T3: ≥7 per week), and logistic regression models were used to estimate odds ratios (ORs) and 95% confidence intervals (CIs) for the association between fish intake and sleep disorders. Low subjective sleep quality and prolonged sleep latency were defined as a sleep quality score of 2 or greater. Low habitual sleep efficiency, short sleep duration, sleep disturbances, daytime dysfunction and use of sleeping medication were defined as a subjective sleep quality score of 1 or greater, subdomains mentioned in previous studies were also applicable in the current study [15,42]. Linear regression models were used to estimate the association between fish intake and scores of sleep quality. Models were adjusted for various factors, including demographic, lifestyle and clinical variables. Model 1 was adjusted for gender, age and BMI. In model 2, we further adjusted for lifestyle factors, including current smoking (yes/no, categories), alcohol consumption (yes/no, categories), salt intake (<6 g/day, ≥6 g/day, categories) and milk consumption (≤1 time per week, 2–6 times per week, and ≥1 time per day, categories). Model 3 represented a comprehensive adjustment for clinical factors, such as the presence of hypertension (yes/no, categories), diabetes (yes/no, categories) and coronary heart disease (yes/no, categories). To assess the consistency of our findings, we conducted additional subgroup analyses stratified by age (<65 years, ≥65 years), gender, BMI (<24 kg/m^2^, ≥24 kg/m^2^), smoking status (yes/no), alcohol consumption (yes/no), hypertension (yes/no), diabetes (yes/no) and coronary heart disease (yes/no). Subgroup analyses were conducted to assess consistency across different demographic and clinical strata. Statistical significance was set at *p* < .05, and analyses were performed using R programming language (version 4.3.0; R Foundation for Statistical Computing, Vienna, Austria).

## 3. Results

### 3.1. Baseline characteristics

**Figure 2.**
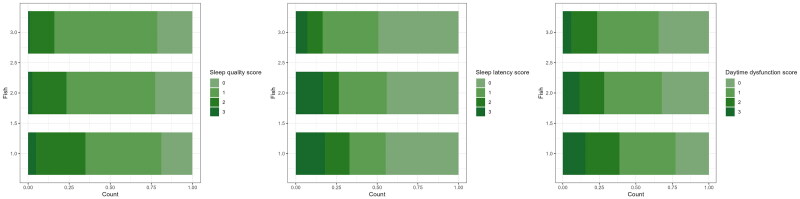
The distribution of subjective sleep quality (A), sleep latency (B) and daytime dysfunction (C) scores across fish intake groups.

Table 1 presents the comprehensive baseline characteristics of the participants, including anthropometric measurements, sociodemographic factors, dietary intake, clinical diseases and sleep parameters, stratified by fish intake frequency. Participants with higher fish intake frequencies were more likely to be male (*p* < .001), younger (*p* = .010), have higher tobacco (*p* = .023) and alcohol intake (*p* = .012), and consume less salt (*p* < .01). Those in the highest tertile of fish intake exhibited lower ISI scores, PSQI scores, indicating better sleep quality (*p* < .001), shorter sleep latency (*p* < .001) and less daytime dysfunction (*p* = .012). Figure 2 shows the percentage of sub-dimensional scores in the three groups.

**Table 1.** Baseline characteristics of study participants by tertiles of fish intake.

Value	Fish intake			
	T1, *n* = 206	T2, *n* = 261	T3, *n* = 437	*p* Value
	(≤1 per week)	(2–6 per week)	(≥7 per week)	
Socio demographics				
Male, %	109 (52.91%)	172 (65.90%)	310 (70.94%)	<.001
Age (≥65 years), %	117 (56.80%)	151 (57.85%)	207 (47.37%)	.010
BMI (≥24 kg/m^2^), %	99 (50.51%)	122 (48.80%)	250 (58.55%)	.026
Lifestyle risk factors				
Alcohol consumption, %	19 (9.45%)	40 (15.69%)	77 (17.87%)	.023
Current smoking, %	33 (16.02%)	71 (27.20%)	109 (24.94%)	.012
Salt intake (<6 g/d), %	76 (36.89%)	71 (27.52%)	100 (23.09%)	<.01
Milk intake, %	182 (88.35%)	227 (86.97%)	373 (85.35%)	.782
Clinical assessment				
Hypertension, %	129 (62.93%)	146 (57.03%)	280 (64.67%)	.131
Diabetes mellitus, %	48 (24.00%)	69 (27.17%)	110 (25.70%)	.363
CHD, %	128 (62.14%)	165 (63.22%)	271 (62.01%)	.947
ISI scores	6.0 [2.0; 10.0]	3.0 [1.0; 8.0]	3.0 [1.0; 7.0]	<.001
PSQI global scores	6.0 [4.0; 10.0]	6.0 [3.0; 9.0]	5.0 [4.0; 8.0]	.021
Sleep disorders %	138 (66.99%)	164 (62.84%)	280 (64.07%)	.441
Low subjective sleep quality, %	72 (34.95%)	61 (23.37%)	70 (16.02%)	<.001
Low habitual sleep efficiency, %	101 (49.03%)	125 (47.89%)	228 (52.17%)	.509
Long sleep latency, %	68 (33.01%)	69 (26.44%)	72 (16.48%)	<.001
Short sleep duration, %	116 (56.31%)	161 (61.69%)	273 (62.47%)	.310
Sleep disturbances, %	191 (92.72%)	237 (90.80%)	398 (91.08%)	.730
Daytime dysfunction, %	159 (77.18%)	177 (67.82%)	287 (65.68%)	.012
Use of sleeping medication, %	10 (4.85%)	9 (3.45%)	15 (3.43%)	.640

Variables using percentages were reported as the Chi-square test among T1, T2 and T3. Other data were reported as Kruskal–Wallis test among T1, T2 and T3. Dietary intake other than fish is based on ≤1 per week.

### 3.2. Association between marine fish intake and sleep disorders

Table 2 displays the ORs and 95% CIs for insomnia, sleep disorders and PSQI components across tertiles of fish intake. Participants with the highest fish intake (≥7 times per week) had a significantly lower likelihood of experiencing insomnia compared to those with the lowest intake (≤1 time per week) (OR: 0.349, 95% CI: 0.242–0.502). This association persisted across all models (models 1–3). Similarly, participants with higher marine fish intake were negatively associated with PSQI subdimension scores for subjective sleep quality (OR: 0.316, 95% CI: 0.205–0.486), sleep latency (OR: 0.462, 95% CI: 0.302–0.706) and daytime dysfunction (OR: 0.568, 95% CI: 0.369–0.861), compared to participants with lower marine fish intake. There was not significant association between marine fish intake and poor sleep quality (OR: 0.959, 95% CI: (0.645–1.417)).

**Table 2.** Odds ratios of sleep disorders or PSQI components and corresponding 95% CIs according to tertiles of fish intake.

Diseases	Fish intake	Cases/participants	Crude model		Model 1		Model 2		Model 3	
			OR (95% CI)	*p* Value	OR (95% CI)	*p* Value	OR (95% CI)	*p* Value	OR (95% CI)	*p* Value
Insomnia	T1	85/206	Reference		Reference		Reference		Reference	
	T2	75/261	0.574 (0.390–0.843)	.005	0.626 (0.419–0.936)	.022	0.567 (0.369–0.868)	.009	0.540 (0.347–0.835)	.006
	T3	86/437	0.349 (0.242–0.502)	<.001	0.382 (0.261–0.558)	<.001	0.333 (0.222–0.499)	<.001	0.318 (0.210–0.479)	<.001
	Per tertile change of fish		0.591 (0.493–0.709)	<.001	0.617 (0.510–0.746)	<.001	0.578 (0.472–0.706)	<.001	0.565 (0.459–0.693)	<.001
Sleep disorders	T1	138/206	Reference		Reference		Reference		Reference	
	T2	164/261	0.833 (0.566–1.222)	.351	0.942 (0.630–1.404)	.769	0.934 (0.610–1.427)	.754	0.905 (0.585–1.395)	.652
	T3	280/437	0.879 (0.617–1.244)	.469	1.000 (0.692–1.440)	.999	0.980 (0.665–1.437)	.917	0.959 (0.645–1.417)	.834
	Per tertile change of fish		0.951 (0.802–1.126)	.562	1.008 (0.843–1.204)	.932	0.997 (0.825–1.202)	.971	0.989 (0.816–1.196)	.906
PSQI component										
Low subjective sleep quality	T1	72/206	Reference		Reference				Reference	
	T2	61/261	0.568 (0.378–0.850)	.006	0.603 (0.395–0.917)	.018	0.549 (0.350–0.856)	.008	0.505 (0.317–0.798)	.004
	T3	70/437	0.355 (0.241–0.521)	<.001	0.397 (0.266–0.590)	<.001	0.326 (0.212–0.498)	<.001	0.316 (0.205–0.486)	<.001
	Per tertile change of fish		0.597 (0.492–0.723)	<.001	0.631 (0.517–0.769)	<.001	0.571 (0.461–0.706)	<.001	0.565 (0.454–0.701)	<.001
Low habitual sleep efficiency	T1	101/206	Reference		Reference		Reference		Reference	
	T2	125/261	0.956 (0.663–1.377)	.807	0.997 (0.678–1.466)	.988	0.961 (0.640–1.441)	.846	1.004 (0.664–1.519)	.985
	T3	228/437	1.134 (0.814–1.581)	.457	1.340 (0.943–1.908)	.103	1.312 (0.909–1.898)	.148	1.331 (0.916–1.939)	.134
	Per tertile change of fish		1.078 (0.917–1.269)	.363	1.178 (0.992–1.401)	.062	1.168 (0.975–1.400)	.092	1.172 (0.976–1.409)	.089
Long sleep latency	T1	68/206	Reference		Reference		Reference		Reference	
	T2	69/261	0.729 (0.488–1.088)	.122	0.841 (0.553–1.281)	.419	0.782 (0.504–1.213)	.273	0.731 (0.464–1.148)	.173
	T3	72/437	0.400 (0.272–0.588)	<.001	0.478 (0.319–0.716)	<.001	0.473 (0.312–0.718)	<.001	0.462 (0.302–0.706)	<.001
	Per tertile change of fish		0.675 (0.554–0.822)	<.001	0.684 (0.560–0.833)	<.001	0.683 (0.556–0.839)	<.001	0.676 (0.548–0.834)	<.001
Short sleep duration	T1	116/206	Reference		Reference		Reference		Reference	
	T2	161/261	1.249 (0.861–1.812)	.241	1.285 (0.873–1.894)	.203	1.183 (0.787–1.780)	.419	1.081 (0.712–1.641)	.716
	T3	273/437	1.292 (0.922–1.808)	.136	1.432 (1.005–2.040)	.046	1.316 (0.907–1.906)	.147	1.225 (0.838–1.788)	.293
	Per tertile change of fish		1.125 (0.953–1.328)	.163	1.187 (0.998–1.413)	.053	1.143 (0.953–1.372)	.150	1.110 (0.922–1.336)	.270
Sleep disturbances	T1	191/206	Reference		Reference		Reference		Reference	
	T2	237/261	0.776 (0.388–1.505)	.459	0.821 (0.407–1.612)	.571	1.115 (0.513–2.410)	.781	1.216 (0.552–2.681)	.624
	T3	398/437	0.801 (0.419–1.460)	.484	0.818 (0.423–1.510)	.533	0.839 (0.420–1.597)	.604	0.875 (0.437–1.674)	.696
	Per tertile change of fish		0.914 (0.677–1.220)	.549	0.917 (0.673–1.235)	.574	0.894 (0.637–1.237)	.507	.907 (0.643–1.261)	.569
Daytime dysfunction	T1	159/206	Reference		Reference		Reference		Reference	
	T2	177/261	0.623 (0.409–0.941)	.026	0.624 (0.401–0.962)	.035	0.676 (0.427–1.062)	.092	0.663 (0.413–1.053)	.084
	T3	287/437	0.566 (0.384–0.823)	.003	0.560 (0.372–0.832)	.005	0.596 (0.390–0.896)	.014	0.568 (0.369–0.861)	.009
	Per tertile change of fish		0.774 (0.645–0.926)	.005	0.771 (0.637–0.931)	.007	0.787 (0.645–0.958)	.018	0.769 (0.627–0.939)	.011
Use of sleeping medication	T1	10/206	Reference		Reference		Reference		Reference	
	T2	9/261	0.700 (0.273–1.769)	.447	0.874 (0.331–2.310)	.782	0.699 (0.240–1.959)	.496	0.566 (0.182–1.642)	.301
	T3	15/437	0.697 (0.311–1.628)	.386	0.974 (0.417–2.399)	.951	0.830 (0.342–2.097)	.683	0.771 (0.318–1.949)	.570
	Per tertile change of fish		0.844 (0.558–1.291)	.424	0.996 (0.648–1.555)	.986	0.925 (0.586–1.477)	.739	0.900 (0.566–1.450)	.659

Model 1: adjusted for age, gender and BMI. Model 2: model 1 plus an adjustment for lifestyle factors (current smoking, alcohol consumption, salt intake and milk intake). Model 3: model 2 plus an adjustment for clinical factors (hypertension, diabetes and CHD).

### 3.3. Association between marine fish intake and the scores of ISI and PSQI

Linear regression analyses revealed a significant negative association between marine fish intake and PSQI total score (*β*: −0.251, 95% CI: −0.434 to −0.069) and severity of insomnia (*β*: −0.451, 95% CI: −0.630 to −0.272). Consistently, total scores for shared items between ISI and PSQI scales showed negative correlations with fish intake (*β*: −0.466, 95% CI: −0.644 to −0.288 for total score 1; *β*: −0.415, 95% CI: −0.595 to −0.235 for total score 2; see Table 3).

**Table 3.** Coefficients of scores of PSQI and ISI and corresponding 95% CIs according to tertiles of fish intake.

	Fish intake	Participants	Crude model		Model 1		Model 2		Model 3	
			*β* (95% CI)	*p*	*β* (95% CI)	*p*	*β* (95% CI)	*p*	*β* (95% CI)	*p*
PSQI scores	T1	*n* = 206								
	T2	*n* = 261	−0.162 (−0.344, 0.020)	.081	−0.119 (−0.304, 0.066)	.207	−0.142 (−0.338, 0.055)	.157	−0.174 (−0.376, 0.028)	.091
	T3	*n* = 437	−0.276 (−0.441, −0.111)	.001	−0.205 (−0.374, −0.037)	.017	−0.226 (−0.405, −0.048)	.013	−0.251 (−0.434, −0.069)	.007
ISI scores	T1	*n* = 206								
	T2	*n* = 261	−0.306 (−0.486, −0.126)	<.001	−0.259 (−0.442, −0.076)	.006	−0.292 (−0.486, −0.098)	.003	−0.322 (−0.520, −0.124)	.001
	T3	*n* = 437	−0.464 (−0.627, −0.300)	<.001	−0.400 (−0.567, −0.233)	<.001	−0.427 (−0.603, −0.251)	<.001	−0.451 (−0.630 − 0.272)	<.001
Total score 1 (ISI)	T1	*n* = 206								
	T2	*n* = 261	−0.269 (−0.449 to −0.089)	.003	−0.210 (−0.393 to −0.028)	.024	−0.233 (−0.427 to −0.039)	.018	−0.274 (−0.471 to −0.077)	.007
	T3	*n* = 437	−0.487 (−0.650 to −0.324)	<.001	−0.425 (−0.592 to −0.259)	<.001	−0.436 (−0.612 to −0.260)	<.001	−0.466 (−0.644 to −0.288)	<.001
Total score 2 (PSQI)	T1	*n* = 206								
	T2	*n* = 261	−0.199 (−0.379 to −0.018)	.031	−0.141 (−0.323 to 0.041)	.129	−0.156 (−0.350 to 0.039)	.117	−0.194 (−0.393 to 0.005)	.056
	T3	*n* = 437	−0.438 (−0.601 to −0.275)	<.001	−0.377 (−0.543 to −0.211)	<.001	−0.382 (−0.558 to −0.205)	<.001	−0.415 (−0.595 to −0.235)	<.001

Model 1: adjusted for age, gender and BMI. Model 2: model 1 plus an adjustment for lifestyle factors (current smoking, alcohol consumption, salt intake and milk intake). Model 3: model 2 plus an adjustment for clinical factors (hypertension, diabetes and CHD).

### 3.4. Subgroup analysis

**Figure 3.**
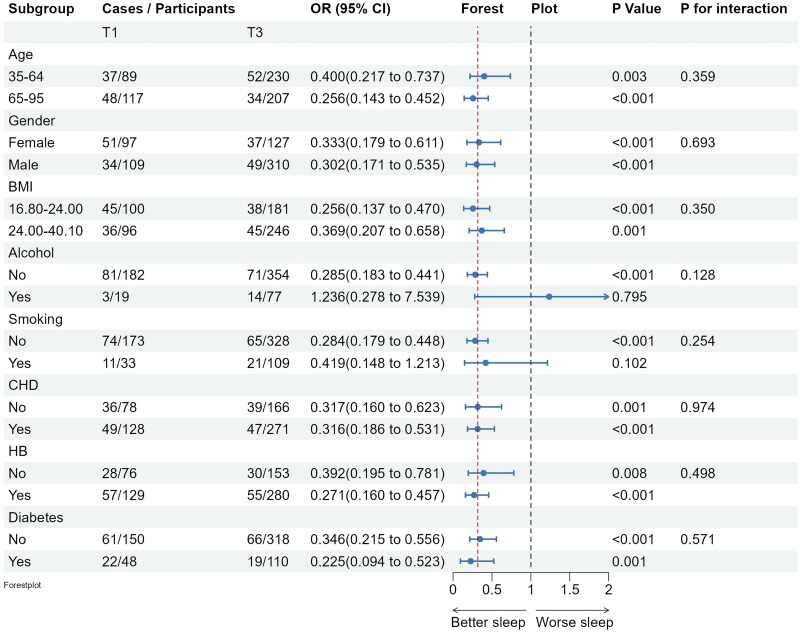
Subgroup analyses of the associations (ORs, 95% CIs) between fish intake and insomnia score among 904 participants. Logistic regression models were adjusted for age, gender, BMI, lifestyle factors and clinical factors, except stratifying factors. The red vertical line on the left refers to the OR value of this dimension for the whole population. BMI: body mass index; CHD: coronary heart disease; HB: hypertension.

**Figure 4.**
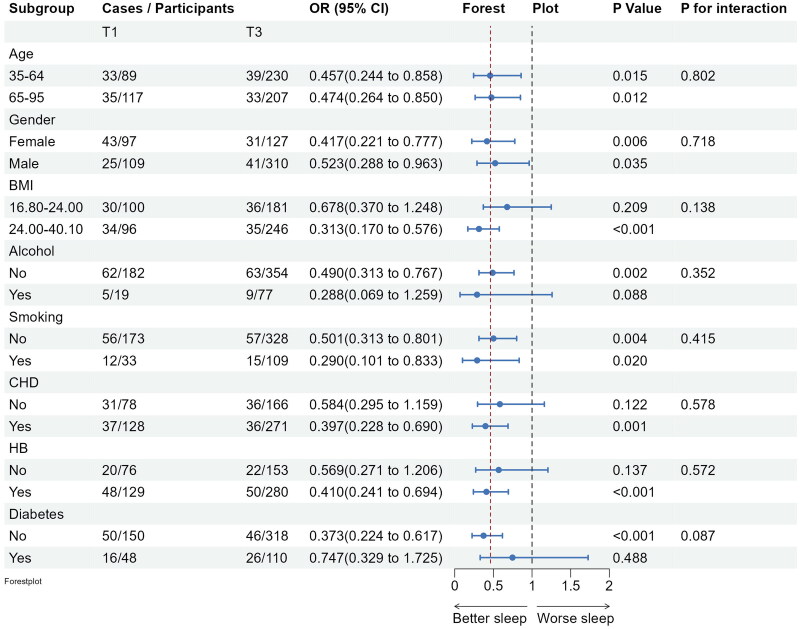
Subgroup analyses of the associations (ORs, 95% CIs) between fish intake and sleep latency score among 904 participants. Logistic regression models were adjusted for age, gender, BMI, lifestyle factors and clinical factors, except stratifying factors. The red vertical line on the left refers to the OR value of this dimension for the whole population. BMI: body mass index; CHD: coronary heart disease; HB: hypertension.

**Figure 5.**
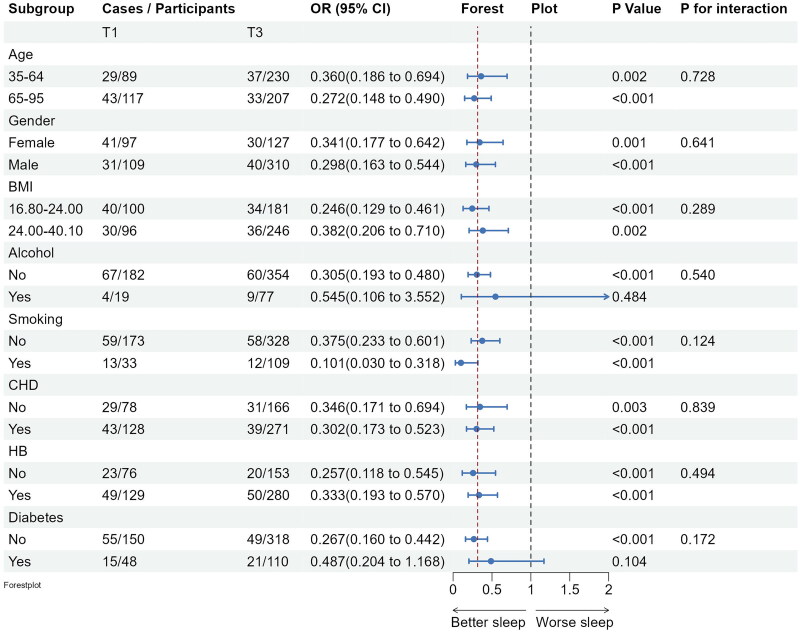
Subgroup analyses of the associations (ORs, 95% CIs) between fish intake and subjective sleep quality score among 904 participants. Logistic regression models were adjusted for age, gender, BMI, lifestyle factors and clinical factors, except stratifying factors. The red vertical line on the left refers to the OR value of this dimension for the whole population. BMI: body mass index; CHD: coronary heart disease; HB: hypertension.

**Figure 6.**
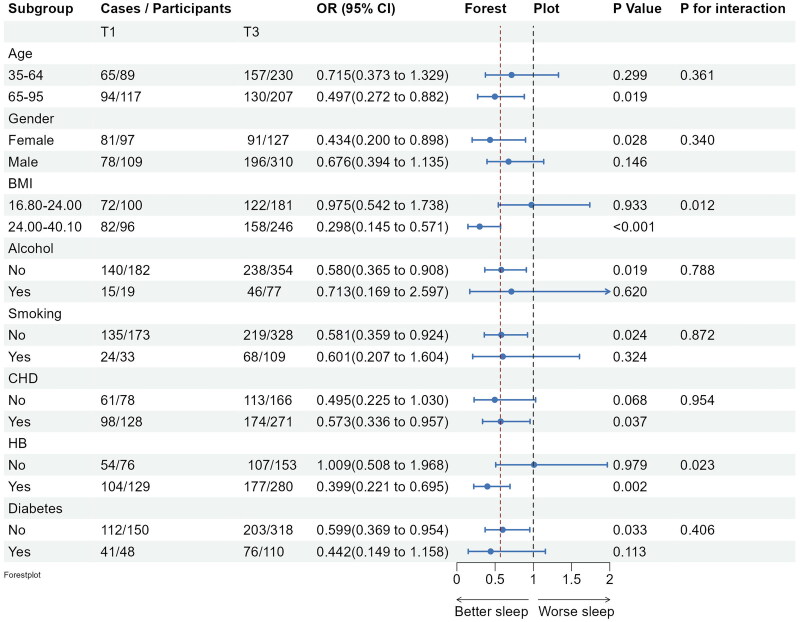
Subgroup analyses of the associations (ORs, 95% CIs) between fish intake and daytime dysfunction score among 904 participants. Logistic regression models were adjusted for age, gender, BMI, lifestyle factors and clinical factors, except stratifying factors. The red vertical line on the left refers to the OR value of this dimension for the whole population. BMI: body mass index; CHD: coronary heart disease; HB: hypertension.

Subgroup analyses confirmed the stability of the logistic regression model across various demographic and clinical factors. Inverse associations between fish intake and insomnia persisted across subgroups, with no significant interactions observed (see Figure 3). Significant negative associations between marine fish intake and several PSQI subdimension scores, specifically, daytime dysfunction, sleep latency and subjective sleep quality scores were observed. Furthermore, significant negative associations between marine fish consumption and several PSQI subdimension scores, such as, daytime dysfunction, sleep latency and subjective sleep quality scores were observed among obese individuals and those with hypertension and coronary heart disease (see Figures 4–6).

## 4. Discussion

In our study, we aimed to investigate the relationship between marine fish intake and sleep disorders, focusing on specific indicators. The present study observed significant negative associations between marine fish intake and several PSQI subdimension scores, specifically, daytime dysfunction, sleep latency and subjective sleep quality scores, suggesting our participants who had higher marine fish intake were more likely to report experiencing better sleep quality, less time needed to fall asleep, and little to no drowsiness/dysfunction the following day compared to participants with lower marine fish intake. Additionally, higher fish intake is associated with lower levels of insomnia severity across the entire population. We also observed a significant negative association between marine fish intake and PSQI subdimension scores for subjective sleep quality, sleep latency and daytime dysfunction among individuals with obesity, coronary heart disease and hypertension.

Our primary finding indicated participants with had higher marine fish intake were more likely to report experiencing better sleep quality, less time needed to fall asleep, and little to no drowsiness/dysfunction the following day compared to participants with lower marine fish intake. Previous studies have reported that increased consumption of vegetables, fruits and fish is associated with better sleep quality [31,48]. Unhealthy eating habits, including low fish intake, have been linked to poor sleep quality [49]. Evidence suggests that increasing fish intake can further enhance sleep quality, even among individuals already consuming more than the recommended amount of fish [31]. Clinical studies have also indicated a positive impact of fish consumption on overall sleep and daytime functioning [14]. Oily fish intake has been linked to decreased sleep latency in healthy schoolchildren [32], corroborating our finding that participants with higher marine fish intake reported less time needed to fall asleep and minimal drowsiness or dysfunction the following day.

The physiological connection between marine fish intake and sleep is well-established. A two-arm, double-blind, randomized, placebo-controlled crossover study investigated the effect of a 4-week fish hydrolysate intervention on sleep in a healthy German population. The supplementation of fish hydrolysate significantly improved subjective sleep quality measured with the PSQI-score and individuals reported improvements in sleep efficiency and a reduction in sleep disturbances and daytime sleepiness during fish hydrolysate intake [50]. Another study assessed the effects of oily fish (>5% fat) consumption on sleep quality in community adults living in rural coastal Ecuador and found oily fish consumption was associated with better sleep quality [31]. In addition, forensic patients from a secure forensic inpatient facility in the USA received Atlantic salmon three times per week over a period of 5 months, which appeared to positively influence both general sleep quality and daytime functioning [14]. The review of diet and sleep mentioned that fatty fish was a good source of vitamin D and omega-3 fatty acids, nutrients that were important for sleep regulation [11].

Marine fish have long been known for their relatively high amounts of melatonin compared to other animal diet, which has therapeutic benefits for sleep disorders, particularly in reducing sleep latency and improving sleep quality [8,29,51]. Melatonin is a potential therapeutic intervention for improving sleep quality in people with autistic spectrum disorder (ASD) [51]. In addition, for adults with respiratory diseases, metabolic disorders and primary sleep disorders, exogenous melatonin supplements were found to be a significant improvement in sleep quality [20]. Another earlier meta-analysis demonstrated that melatonin decreases sleep onset latency, increases total sleep time and improves overall sleep quality in adults and children diagnosed with primary sleep disorders [52]. Furthermore, in a double-blind, placebo-controlled study, participants with either normal sleep (*n* = 15) or confirmed reduced sleep efficiency based on actigraphy (*n* = 15) received placebos and three doses of melatonin (0.1, 0.3 and 3.0 mg) orally in random order 30 min before bedtime for one week, melatonin reduced the time to sleep and improved the quality of sleep, even in normal people without significant sleep disorders [53]. Although marine fish contain a relatively high amount of melatonin in their diet, approximately 3.7 ng/g [16], this concentration remains low compared to over-the-counter melatonin supplements available on the market, which typically contain 0.5–3 mg per tablet or serving.

Vitamin D, regulates the sleep–wake cycle by affecting areas of the brain involved in sleep regulation [54,55]. Recent studies have suggested a positive impact of fish intake on sleep quality and daytime functioning, potentially attributed to its role in maintaining adequate vitamin D levels, especially in populations with limited sunlight exposure [14,30]. Omega-3 fatty acids, particularly EPA and DHA found in fish, regulate neurotransmitter synthesis and release in the brain, influencing sleep patterns [14,56].

Furthermore, inflammatory cytokines may serve as an underlying mechanism in the association between fish consumption and sleep quality. Components present in fish, such as collagen hydrolysate, omega-3 fatty acids and vitamins, may attenuate inflammation involved in sleep regulation, including TNF-α, IL-6 and IL-1β. Elevated levels of IL-1β and TNF-α have been linked to various sleep disorders, such as sleep apnoea and chronic insomnia [57–59].

In relation to this study, relatively few empirical research has been conducted on the Chinese population regarding the relationship between fish intake and sleep. Evidence showed that higher consumption of fish, shellfish and mollusks was correlated with a lower risk of poor sleep in Chinese urban adults from eight cities [60]. Additionally, in a study conducted on Chinese schoolchildren aged 9–11 years, frequent consumption of fish was independently associated with less sleep disturbances, which indicated better overall sleep quality [61]. Furthermore, a dose–response relationship indicated less sleep disturbances in schoolchildren who had higher fish consumption (always, 4.49 points and sometimes, 3.01 points), compared to those who rarely ate fish. The present study extends upon previous observations regarding the relationship of fish-intake and sleep among the Chinese population; and discovered an association between overall sleep quality and marine fish intake among Chinese cardiovascular patients, a finding similar to previous Western studies [62–64]. Thus, the present study strengthens the speculation that fish intake is beneficial for sleep quality.

Our study also revealed the association between fish diet and some different dimensions of sleep among male and female patient populations. Significant differences in fatty fish consumption frequency between male and female patients were observed in this study; wherein male patients reported a higher fatty fish consumption frequency score (*M* = 3.56) compared to female patients (*M* = 3.18), which varied greatly between different nations (Supplementary Table 1) [65–67]. Among individuals with coronary heart disease, hypertension and obesity, participants who had higher marine fish intake were more likely to report experiencing better sleep quality, less time needed to fall asleep, and little to no drowsiness/dysfunction the following day, which was consistent with previous findings. Fish is renowned for its rich content of fatty acids, which can ameliorate sleep disorders stemming from abnormal lipid metabolism in individuals with coronary heart disease [68]. A cross-sectional study demonstrated that dietary supplementation with DHA-rich fish oil reduced heart rate and enhanced heart rate variability (HRV), thus improving parasympathetic–sympathetic balance in overweight adults at risk for coronary artery disease [69]. Interestingly, improved sleep latency may be linked to heightened general arousal levels and reduced parasympathetic activity [14]. Additionally, fish consumption has been shown to induce feelings of sleepiness in the evening, leading to improved sleep and executive functioning during the day [14], which aligns with our findings. Evidence suggests that fish intake can alleviate sleep disturbances associated with arterial hypertension in hypertensive patients [70]. Our results also revealed that people with higher marine fish intake were more likely to report experiencing better sleep quality, less time needed to fall asleep, and little to no drowsiness/dysfunction in the hypertensive population. Polyunsaturated fatty acids in fish can enhance vasodilation and arterial compliance by reducing angiotensin-converting enzyme (ACE) activity and activating the parasympathetic nervous system, factors linked to sleep and wakefulness [71,72]. The positive association between fish consumption and sleep may be attributed to the effects of fatty acids on cardiac/autonomic nerves and blood vessels, particularly in hypertensive individuals [14,73]. Furthermore, in obese people, we also found similar ameliorative effects of marine fish on sleep. There is mounting evidence linking obesity to poor sleep quality [74]. Moreover, dietary supplementation with DHA-rich fish oil has been shown to improve the parasympathetic–sympathetic balance in obese individuals, potentially impacting sleep latency [69]. Recent studies suggest that inflammatory factors and neuromodulators such as serotonin, norepinephrine, prostaglandins, adenosine and sleep-regulating peptides may be mechanisms linking obesity to poor sleep quality, with vitamin D in fish potentially playing a role in improving sleep quality by influencing these factors in obese individuals [57,58,75].

While our study has strengths such as investigating the effects of fish on specific sleep disorder indicators and adjusting for known risk factors, it also has limitations. First, being a cross-sectional study, we may not establish causal associations between fish intake and sleep quality. Second, subjective assessment of sleep quality may introduce recall bias. Third, the semi-quantitative nature of the FFQ used may have affected the accuracy of quantifying fish intake and its associations with sleep quality. The FFQ only asked participants the amount of ‘fish’ consumed without further inquiries into the species of fish consumed. As such, we regret to inform that we are not entirely confident that the fish examined is fatty fish. However, given the location of the research (Wenling), we speculate the fish examined in this research is most likely fatty fish [76,77]. Finally, the results of our study might due to the special population of Taizhou, Zhejiang, China, where marine fish intake may be relatively high. Therefore, fish may be more beneficial to the sleep quality of people in coastal areas than in other areas.

In conclusion, our findings indicate that higher fish intake is associated with lower daytime dysfunction, sleep latency and sleep quality scores. Furthermore, we observed a positive and stable association between fish intake and sleep quality in individuals with obesity, coronary heart disease and hypertension. These findings suggest that fish may serve as an effective natural aid for sleep-related disorders [11]. However, further investigation is warranted to elucidate the specific underlying mechanisms responsible for the beneficial effects of fish consumption.

## Supplementary Material

Supplementary Tables.docx

## Data Availability

The data that support the findings of this study are available from the corresponding author, Y. Liu (email: benjaminlyl@wmu.edu.cn), upon reasonable request.
