# Supplementary material for: Association between fish consumption and sleep disorders among Chinese adults: a cross-sectional study
Source: Ann Med. 2025 Apr 20;57(1):2491663. doi: 10.1080/07853890.2025.2491663 (PMC12013145; doi:10.1080/07853890.2025.2491663)
Supplement: Supplementary Tables.docx [file IANN_A_2491663_SM4343.docx]

- Supplementary Table.1: Significant differences in fatty fish consumption frequency between male and female patients.

| **Characteristic** | **Female, N = 313** | **Male, N = 591** | **p-value** |
| --- | --- | --- | --- |
| **Marine fish** | 3.18 (1.23) | 3.56 (1.25) | <0.001 |

The data is represented as mean (standard deviation) and the statistical method used is t-test.

- Supplementary Table.2: Significant differences in fatty fish consumption frequency between male and female patients in different groups.

|  |  | **Female** | **Male** | **p-value** |
| --- | --- | --- | --- | --- |
| **T1 (female = 97, male = 109)** | **≤ 1 per week** | 1.69 (0.46) | 1.53 (0.50) | 0.019 |
| **T2 (female = 89, male = 172)** | **2-6 times / per week** | 3 (0) | 3 (0) | null |
|  |  |  |  |  |
| **T3 (female = 127, male = 310)** | **≥ 7 per week** | 4.45 (0.50) | 4.59 (0.49) | 0.007 |

The data is represented as mean (standard deviation) and the statistical method used is t-test.

- Supplementary Table.3: Differences in number of male and female patients for the different tertiles.

|  |  | **Female** | **Male** | **p-value** |
| --- | --- | --- | --- | --- |
| **T1(n=206)** | **<1 time / per month (1)** | 89 | 103 | 0.4 |
|  | **<1 time / per week (2)** | 8 | 6 |  |
| **T2(n=261)** | **2-6 times / per week (3)** | 89 | 172 |  |
| **T3(n=437)** | **1 time / per day (4)** | 70 | 127 | 0.007 |
|  | **≥ 2 times / per day (5)** | 57 | 183 |  |

The statistical method used is the chi-square test.

- Supplementary Table.4: Differences in number of male and female patients in all population.

| **Characteristic** | **Female, N = 313** | **Male, N = 591** | **p-value** |
| --- | --- | --- | --- |
| **Marine fish** |  |  | <0.001 |
| **1** | 30 (9.6%) | 51 (8.6%) |  |
| **2** | 67 (21%) | 58 (9.8%) |  |
| **3** | 89 (28%) | 172 (29%) |  |
| **4** | 70 (22%) | 127 (21%) |  |
| **5** | 57 (18%) | 183 (31%) |  |

Categorical data are presented as numbers and percentages. The statistical method used is the chi-square test.
